# The Need for an Evidence-Based Program in Sweden to Support Parents to Create Healthy Lifestyle Behaviors from the Start of Life—Parental Perceptions

**DOI:** 10.3390/nu12123823

**Published:** 2020-12-14

**Authors:** Maria Henström, Ulrika Müssener, Karen J. Campbell, Kylie D. Hesketh, Magdalena Rosell, Christine Delisle Nyström

**Affiliations:** 1 Department of Biosciences and Nutrition, Karolinska Institutet, NEO, Group MLÖ, 141 83 Huddinge, Sweden; maria.henstrom@ki.se (M.H.); magdalena.rosell@ki.se (M.R.); 2 Department of Health, Medicine and Caring Sciences, Division of Society and Health, Linköping University, SE-581 83 Linköping, Sweden; ulrika.mussener@liu.se; 3 Institute for Physical Activity and Nutrition (IPAN), Deakin University, Geelong 3125, Australia; karen.campbell@deakin.edu.au (K.J.C.); kylie.hesketh@deakin.edu.au (K.D.H.)

**Keywords:** child healthcare, feeding, infant, intervention, physical activity, qualitative research, screen time, thematic analysis

## Abstract

In Sweden, there have been no interventions promoting the development of healthy lifestyle behaviors starting in infancy. Thus this paper aims to: (i) investigate Swedish parents’ experiences regarding feeding of solid foods, screen time, and physical activity in healthy infants; (ii) investigate parents’ needs and perceptions regarding information/support provided in primary child healthcare (CHC) and (iii) explore whether a parenting program focused on child diet and active play would be relevant and utilized. Semi-structured interviews were conducted with 20 parents. These were recorded, transcribed verbatim and analyzed using thematic analysis. Seven themes emerged: Feeling excited to enter a new phase; Parents’ responsibility of doing it “right” can be stressful; Motivated to learn during specific time windows; Information to trust; The importance of social support from peers; Experiences of support received from CHC; and the Infant, Feeding, Activity, and Nutrition Trial (INFANT) for a Swedish context. Parents were excited to enter new phases in their child’s development. However, this came with worry they were doing things “right”, leading parents to want more information/support. Overall, Swedish parents were very positive about the prospects of engaging in a primary CHC delivered program to support them to promote healthy lifestyle behaviors from birth and felt this could complement the care currently provided.

## 1. Introduction

Childhood overweight and obesity is a serious health challenge and commonly stems from unhealthy lifestyle behaviors (e.g., diet, physical activity, and sedentary behavior). Globally, approximately 38 million infants and young children (i.e., 0–5 years) live with overweight or obesity [1]. In Sweden approximately 12% of four year olds were classified as having overweight or obesity in 2018 [2,3], with this figure almost doubling when children reach 10 to 11 years of age [4,5]. To date in Sweden and the European Union, several well-conducted obesity prevention interventions in pre-school aged children have been conducted; however, limited [6,7] or no effects [8] regarding lifestyle behaviors have been achieved. As lifestyle behaviors are established prior to pre-school age [9,10,11], there is a great need to focus on primary prevention from the beginning of life.

The first 1000 days (i.e., conception to 24 months) has been found to be the time point of greatest developmental plasticity and thus the period with the maximum potential to influence health throughout life [12]. Compared to other age groups such as pre-school and school-aged children, to date, there have been very few interventions focusing on infancy (i.e., from 0–2 years). In a systematic review by Redsell et al. [13], which included 27 randomized controlled trials aiming to reduce the risk of overweight or obesity directly or indirectly in infancy, it was found that most of the multi-component interventions demonstrated positive effects regarding a component of diet or responsive feeding. However, very few of the interventions included components to promote physical activity and reduce sedentary behavior [13].

The implementation of promising interventions in new contexts can spare financial and human resources related to the development processes [14]. To the best of our knowledge in Sweden, there have been no interventions promoting the development of healthy lifestyle behaviors starting in infancy. As the prevalence of overweight and obesity amongst Swedish children has doubled and quadrupled in the past three decades, respectively [4], there is a need for an evidence-based program to support parents to embed healthy lifestyle behaviors from the start of life. On reviewing interventions for a Swedish setting, we identified the Melbourne Infant, Feeding, Activity, and Nutrition Trial (INFANT) as a program that could potentially be transferred. INFANT was a multi-component intervention delivered through primary child healthcare (CHC) in Victoria, Australia, that showed some promising outcomes regarding diet and television viewing [15,16] which were sustained to five years of age [17]. The intervention, now delivered at scale, consists of four group sessions led by a health professional [18], starting when the infant is approximately four months of age and running through the first year of life. The group sessions consist of approximately 4–10 parents with children of similar age and aim to build parental knowledge, skills, and social support with regards to infant feeding, physical activity, and screen time [15,16]. In addition, group sessions are complemented by a mobile app, which contains information on all of the health behaviors, as well as providing tips and strategies that enhance the information provided in the group sessions. The app is referred to in the group sessions and parents use it at their own discretion.

As Swedish primary CHC is similar to Australian CHC with regards to healthcare settings and opportunities, INFANT or a similar program may be an ideal complement to existing care. More specifically, in Sweden parents are assigned a CHC nurse when their child is approximately one week old and between 3–12 months of age there are five scheduled visits at regular increments [19]. Furthermore, all parents are invited to attend a parental group at their primary CHC when their child is between 6–12 weeks old. The overall aim of these groups is to offer parents a social network, increase parents’ knowledge with regards to child development and parent/child relationships for example, as well as provide parents the opportunity to reflect on their parenting in groups [2,19].

Before a program such as INFANT is tested in Sweden it has to be carefully adapted to fit the new target population. Therefore, the aims of this paper are to: (i) investigate Swedish parents’ experiences regarding feeding of solid foods, screen time, and physical activity in healthy infants; (ii) investigate parents’ needs and their perceptions of what information and support is provided in primary CHC in Sweden, and (iii) explore whether a program like INFANT would be relevant and utilized by Swedish parents.

## 2. Materials and Methods

### 2.1. Participants and Recruitment

Participants for this study were recruited using convenience sampling. Our personal networks made contact with parents they knew with an infant aged 2–12 months. Parents who consented to their contact information being shared with the research team were then sent the full study information and formal consent via email. Further snowball recruitment from these parents also occurred. Inclusion criteria for this study were: (i) parents needed to have an infant aged between 2–12 months at the time of recruitment and (ii) be able to speak and read Swedish sufficiently well in order to participate in a semi-structured interview and provide informed consent. There were no exclusion criteria.

A total of 23 parents were invited to participate. Three parents declined to take part in the interviews (one stated lack of time and two did not respond to the invitation email) leaving a total of 20 participants. All participants resided in Stockholm County or the County of Östergötland in Sweden. One parent from each family was asked to partake in the interviews and the choice of who participated was made by the parents. For all participating families one of the parents was on parental leave at the time of the interview.

Once informed consent was obtained, telephone interviews were scheduled at a time convenient for the participating parent. The participating parent also filled in a brief one-page questionnaire providing basic demographic information on themselves and their infant which was sent via post. All data collection took place in May/June 2020. All participants provided their informed consent before the interviews took place and the study was conducted in accordance with the Declaration of Helsinki. This study follows the COnsolidated criteria for REporting Qualitative research (COREQ) checklist (Scheme S1) [20], and the Swedish Ethical Review Authority (2020-00814) approved this study.

### 2.2. Semi-Structured Interviews

All interviews were performed once over the telephone in Swedish by M.H., and the only people present were M.H. and the interviewee. At the beginning of each interview, consent to participate and to record the interview (audio) were reconfirmed. Thereafter, the participant received a brief overview of the aims of the study. The same set of core questions were asked to each participant (see Table S1), with follow-up questions tailored to individual responses using a semi-structured format. The core questions were constructed and critically discussed by the research team (all female) who are experienced in obesity prevention interventions focused on promoting healthy lifestyle behaviors in the early years. Three of the researchers are nutritionists (M.H., M.R., and C.D.N.), one is an occupational therapist (U.M.), and K.J.C. (a dietician) and K.D.H. are child public health researchers who developed INFANT. The backgrounds of the researchers (all have PhD’s) were made clear as data interpretation can be influenced by this. The core questions were divided into four topics: (i) parents’ experiences with introducing food to their infant; (ii) roles and responsibilities regarding children’s food and movement behaviors; (iii) information and support that parents received or would have liked to receive; and (iv) adaption of a program such as INFANT for a Swedish context. Furthermore, before topic four the participants received a very short summary of INFANT. This short summary consisted of how INFANT was set-up (i.e., four group sessions provided through primary CHC complemented by a mobile app) and that it begins when the infant is around four months of age. Note, INFANT was not mentioned to participants prior to topic four to ensure no influence on responses to the first three topics. All interviews were audio recorded and transcribed verbatim using a professional transcribing service and the transcripts were not returned to participants. On average the interviews were 55 min in duration (range: 43 to 73 min), and directly after each interview, reflexive notes were written by M.H. Reflexive notes were written as a way of documenting contextual information directly after the interview and these notes included observations regarding interactions as well as the interviewer’s impressions.

### 2.3. Data Analysis

The paradigm used in this study was constructivist with an interpretive method [21] and the interview transcripts were analyzed using thematic analysis [22,23]. All interviews were read by C.D.N. and M.H. who are post-doctoral research fellows and coded by M.H. using an inductive approach (i.e., data driven) [22,23] using Microsoft Excel 2016 (Microsoft, Redmond, WA, USA). Microsoft Excel 2016 was used to organize the data and to subjectively create codes based on the interpreted meaning of the respondents’ statements. The analysis followed a prescribed process following Braun and Clarke’s guidelines for thematic analysis [22]. The analysis was done by M.H. and involved C.D.N. and U.M. through continuous discussions to achieve a shared understanding of each theme. The codes used were developed during an iterative process of coding and from what the interviewees talked about. The analysis process was done under the guidance of U.M. who has extensive experience working with qualitative data. These researchers also led the development of the core questions. If questions or disagreements arose regarding the interpretation of the themes K.J.C., K.D.H., and M.R. were brought in to discuss the interpretation. Finally, during the analysis saturation was reached where no new codes or themes were identified. The original Swedish interviews were utilized in the analysis and the quotations used to illustrate the themes were translated into English. IBM SPSS statistics version 25 (IBM, Armonk, NY, USA) was used to analyze the demographic variables using descriptive statistics.

## 3. Results

Table 1 provides the descriptive characteristics of the participating parents and infants. Overall, there was a large variation in the participating infant’s age as well as parental age and body mass index (BMI). The majority of the participating parents were female, had a university or college education, and were born in Sweden. According to BMI, 14 parents (70%) were classified within the healthy weight range, 5 parents (25%) in the overweight range, and 1 parent (5%) in the obese range.

**Figure 1:**
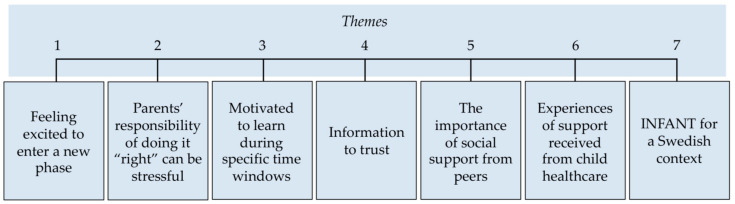
Themes identified during thematic analysis.

During the thematic analysis of the interviews seven themes were developed (Figure 1). Overall, parents expressed a need for more information and support. Even though the interviews included questions regarding food, physical activity, and screen time the majority of parents tended to focus their discussion around food. Each quote has been labeled with M for mother or F for father plus a unique identifying number as well as the infants’ gender and age.

### 3.1. Theme 1: Feeling Excited to Enter a New Phase

When the interviewed parents were asked to describe their experience and feelings around introducing solid foods to their child, they most often first described this as exciting or fun. They were looking forward to see their child’s reactions when trying out new foods, and felt excited to start introducing solid foods, especially if their infant had already started to show interest in it. Parents also described a satisfactory feeling of moving forward into the next phase of their child’s development—food introduction was considered a “big step”.

“It was like a new step in the development, something new that he will experience and me too.”

(Mother 2, boy 2mo)

For some families, this change also came with welcomed advantages; for instance, the partner would now be able to take a bigger role around feeding, especially if the mother had been exclusively breastfeeding until then.

“I think it’s going to be fun. It will be exciting. A bit of fun. // In part, it will be fun because I can take on a bigger role. Now it’s exclusive breastfeeding for him.”

(Father 3, boy 3mo)

“Very positive. Mainly, because I am exclusively breastfeeding and she does not want to take a pacifier or a bottle, so it [food introduction] gives my partner the opportunity to step in a little more, in a different way.”

(Mother 14, girl 2mo)

### 3.2. Theme 2: Parents’ Responsibility of Doing It “Right” Can Be Stressful

When introducing solid food between 4–6 months, positive feelings described by parents were also accompanied by stress and uncertainty. The very first thing most parents mentioned, when asked about their responsibility around food, was to ensure that the child gets what he or she needs—in terms of nutrients, variety and amounts. However, this is where parents also felt anxious, especially if their child was perceived as a picky eater or simply did not eat “enough” of the food served.

“Now he eats very well, knock on wood, but I mean if he would sort of stop eating as well as he does now, then it would be hard. Now we still get him to eat what we serve.”

(Mother 9, boy 7mo)

Some of the parents spoke about the challenge of practicing a responsive feeding approach and not pressuring the child to eat despite their concerns around the child not eating enough. At the same time, most parents felt unsure regarding appropriate portion sizes for infants. When food introduction was discussed, the challenge of “translating” breast milk to solid foods was often highlighted: parents considered it difficult to know how much food corresponds to the same amount of energy provided through breastfeeding, and how to make sure important nutrients were provided and consumed. Some parents specifically raised the issue of not being able to verify whether nutritional needs, such as iron, were sufficiently met or not—and this created stress.

“There were no fixed routines and times [when breastfeeding], then all of a sudden you have to start [introducing food]. Well, is it breakfast, lunch, dinner, or what is it … and when? When and how much? It was … I thought it was a bit…it was hard [food introduction].”

(Father 1, boy 10.5mo)

Another aspect of parents’ perceived responsibilities related to understanding child feeding behaviors and hunger cues, and being able to respond and adjust strategies depending on the child’s needs. In other words, parents felt they were expected to learn how to listen to their child. This responsibility was brought up as a great challenge by several parents, who pointed out that every child is different and that it can be difficult—especially as a first-time parent—to learn how to “read” their own child and know how to act in response to his or her cues. For instance, parents could describe different concerns depending on the situation: “my child does not want to eat; what can I do?” or “my child loves food; how much can I serve at one meal and when do I increase the amount?” Thus, feeding advice given from primary CHC or other information sources that include messages such as “try and see what works” or “see what the child thinks” were often perceived by the parents as difficult to interpret and put in practice.

“… and it always is like this ‘yes, and then you have to start from what your child thinks’, but it is also difficult, because it is not easy to know exactly what your child thinks.”

(Mother 15, girl 6.5mo)

Furthermore, parents highlighted the need for more concrete advice that simplifies a parent’s engagements with their child. They mentioned that advice should be feasible to implement in a busy family life and helpful in easing concerns and lowering the stress around mealtimes, for example by providing useful feeding practice advice and simple tips on which foods to serve. Overall, it was considered important to take into account the well-being of the whole family, especially considering how the first year as a parent often can be perceived as difficult.

“It’s about making good food, but it’s also about it being good for all of us, that is, that we [the family] should feel good. Eh, and do not put too much pressure on ourselves. // It’s not just about food, it’s about the balance in general.”

(Mother 4, boy 12mo)

Beyond food and feeding, screen time for children was regarded as a sensitive issue. Parents felt surrounded by controversial opinions and expressed uncertainty regarding what type and how much screen time is considered “healthy”, which could be stressful. Most parents seemed to have discussed and reflected upon screen time and had ideas before becoming a parent on how much to restrict it. However, often these ideas had changed once they became parents. Different “rules” seemed to exist in different families, and even though parents seemed concerned about allowing too much screen time, many also described daily life situations where screens were allowed and “needed”. Different types of screen time were also discussed, where some parents considered it more acceptable if it had a pedagogic purpose. In addition, the digitalization of the world was discussed as something that affects peoples’ use of screens today and thereby, in the parents’ opinion, makes screen time unavoidable even in early childhood.

“I had an idea on it [screen time] … not to introduce it, not to have it. But it disappeared very quickly.”

(Mother 8, boy 9.5mo)

“… She gets calmer when there’s a little TV or Babblarna [child’s TV program] on. But most of all, I wish she had no screen time now at all. // I do not know what the recommendations are, actually. But I think it should be none for such small children. But yes, we just do what we think.”

(Mother 13, girl 7mo)

### 3.3. Theme 3: Motivated to Learn during Specific Time Windows

It became evident from the interviews, that during the period of food introduction parents felt like “a sponge”—extremely open to receiving information and clearly expressing a need for support. Parents expressed strong beliefs that their actions now could have a great influence on their child’s future health and habits, in terms of food, physical activity, and screen time. Several parents had reflected upon this and took this period as an opportunity to change or introduce new healthy habits to benefit the whole family, such as including more vegetables in their meals.

“… We have also started to think of how we eat now. // We still want to create, uh, some pattern that becomes, uh, her habits, and her picture of how to eat.”

(Mother 14, girl 2mo)

It was deemed important to try to be a good role model and to show and introduce the child to healthy eating and activities with a hope of it becoming a natural part of the child’s habits later in life. Many parents did express that they felt they have a central role influencing physical activity through introducing and encouraging organized sports in the coming years. However, at the present time they were not concerned about physical activity as they considered their child to be “naturally active” at this age. Instead, the majority of the discussion focused around food as parents felt this was more important now.

“And it is not so easy [with food], especially when it is the first child as well. It is not easy to know what is right and wrong.”

(Mother 11, girl 4.5mo)

As described above, new parents felt responsible for providing an optimal nutritional start for their infant. Their drive to do the right thing produced a strong motivation to learn, as they described themselves as feeling inexperienced—“everything is new”—and having “a thousand questions” as first-time parents. However, it became clear from the interviews that there are specific time windows when parents need and seek information. The first months of parenthood were typically described as divided in distinct and intense phases, and parents explained how they were eager to learn but felt completely focused on the present and were therefore most receptive to information relevant now or soon to be of relevance. For example, most parents stated that information on food introduction (e.g., a workshop at primary CHC) should be offered at four months of age or slightly before, i.e., when you are about to start solids; whereas two months was considered “too early” and six months “too late”.

“… You are so into what is happening right now when the baby is so small …”

(Mother 10, boy 10mo)

“… When they are two [months] then it is very long until they are four [months] and there is so much that happens every day. Then you have time to forget it in some way.”

(Mother 16, girl 3.5mo)

“Mm. So for me it feels like after six months, then it feels almost a little too late for me.”

(Mother 6, boy 10mo)

“So like this, ‘Now this phase can come. Be prepared for this to come’ [Wants information from primary CHC in advance]. // Because it … there will be a lot of Google searches, you know.”

(Mother 9, boy 7mo)

### 3.4. Theme 4: Information to Trust

When parents searched for answers regarding their child and food they most commonly reported using the Internet, followed by asking friends with children. A few parents mentioned the CHC nurse; however, it was also pointed out that primary CHC visits occur less frequently as the child grows, and some parents felt like there was not enough time at the visits to ask questions.

When utilizing the Internet, only a few websites (e.g., the National Food Administration) were considered reliable according to parents. Those who visited these websites found them useful when looking for facts, such as “what should be avoided in an infant’s diet?”, although not as useful for tips and inspiration. Instead, parents described how they often end up on discussion forums or social media (e.g., Facebook groups) for these things. However, all interviewees clearly stated that whenever they read something online they were always cautious and took it with a grain of salt, especially as statements can be written by “anyone”.

“… I am pretty careful not to believe everything I read, I need to take it with a grain of salt. But I think it is fun to read about others experiences as well, you take what makes sense [from others’ posts on social media or Internet forums]”.

(Mother 3, boy 9.5mo)

Furthermore, the majority of parents reported using mobile apps for information. Those mentioned, however, were all pregnancy apps and only a couple of them continued after delivery, focusing primarily on child development. Parents reported appreciating the apps, and that learning about different phases and recognizing their own situation in the text created a reassuring feeling. However, none of them had found or used any app focusing on infant food or feeding. Altogether, the parents often turned to the Internet to find answers regarding their child and food; however, conflicting information and lack of trustworthiness in what they found was considered an issue.

### 3.5. Theme 5: The Importance of Social Support from Peers

Even though the Internet seemed to be the primary source of information, it was evident from the interviews that social peer support was extremely important, especially to first-time parents. This seemed to be grounded in parents’ perceived obligation of doing things right for their child and an uncertainty of how to do that, which created a great need to exchange experiences and information with other parents.

“… You want to exchange information with as many [parents] as possible at around four months [tasting portions begin at this time], because you feel … especially with the first child, I think that you are like a piece of blank paper.”

(Mother 9, boy 7mo)

By turning to their peers, parents felt supported and less alone in challenging periods. They could compare development and behaviors between children, which helped in understanding their own child better and could often ease concerns around being on the right track. Seeking peer support also meant creating an idea of how other parents handled specific situations, and was described as a way of getting useful and concrete advice, such as tips on what foods to serve.

Some of the interviewees described their own mom as an important support; however, others explained how they would consciously avoid asking their own parents for advice, especially around feeding, as advice from them may not comply with current guidelines. In their view, this was a cause of different cultures and generations. One interviewee, whose parents were born outside of Sweden, exemplified this with honey—a food commonly given to infants in several countries but avoided before one year of age in Sweden. Others highlighted generational differences and described how their own parents’ feeding strategies, such as pressure to eat, used to be different from what is recommended nowadays.

“I remember from when I was little, it was like, ‘but one more bite, please, a bite for Mom’.”

(Mother 8, boy 9.5mo)

Furthermore, social peer support was not only received from already established friends or family, but also through new contacts. Parents who did not have friends with young children, instead sought that type of support in other places and expressed a stronger motivation to attend first-time parent groups at primary CHC. Overall, parents with experience from parent groups saw them as a great opportunity to meet other parents, and possibly new friends.

“I think the advantage … [with parent groups] first it’s definitely to be able to share other people’s experiences, and to get support. We have and still have a lot of support from each other, I think [participant’s parent group]. We created a group on Facebook where we ask questions about for example regarding preschool and sort of ‘how do you think about this situation’ and stuff like that.”

(Mother 4, boy 12mo)

### 3.6. Theme 6: Experiences of Support Received from Child Healthcare

Parent’s experiences with primary CHC across the first year of life varied greatly. Some of the parents described how they got along very well with “their” CHC nurse and therefore felt comfortable and well supported; others instead expressed disappointment and were left seeking additional information and opportunities to discuss their child elsewhere.

“… I’m so glad we feel comfortable with them [CHC nurses] too, because I know not everyone does. It depends on which nurse you get as a contact person.”

(Mother 4, boy 12mo)

When asking the parents about their experiences with primary CHC, they spoke about having to ask “the right” questions to obtain information. Some parents wrote lists with specific questions to bring to each routine visit with the CHC nurse, whereas others did not and felt insecure about what they should ask about or simply did not remember to ask at the time of the visit.

“It is not always easy to come up with your own questions.”

(Mother 13, girl 7mo)

With regards to screen time for young children, none of the parents were familiar with any recommendations and only four could recall speaking about or at least mentioning screen time at primary CHC. When parents were asked about support from primary CHC around physical activity and active play, the majority mentioned advice on infant tummy time; other than that, additional information and recommendations or advice on how to stimulate physical development were not recalled. In general, it was clear that parents perceived primary CHC as needs-oriented i.e., routine visits aimed at monitoring the child’s development and health, and with information and support provided if a need was identified. It was believed that primary CHC had good competence in children’s physical development, and parents trusted their nurse to react if anything deviated from the normal. Nevertheless, the parents expressed a desire to receive more information and advice around these subjects regardless of if their child was developing “normally”. For instance, they expressed a desire for more information on how to help stimulate physical development by introducing new activities or play.

“… But I have only heard ‘it is normal; it is normal’. Not like ‘it’s normal, but to simulate [development] you can do this’. I would have liked that.”

(Mother 15, girl 6.5mo)

Participation in parent groups had been offered to several parents but not all, with the majority of parents reporting the groups as “more social than informative”. Furthermore, the structure of the parent groups organized through primary CHC seemed to vary greatly, even within the same CHC center.

“I talked to someone who had another CHC nurse at the same CHC center, but they had six sessions instead of the three I had [parent group sessions]. And then they had a theme for each session, with prepared information before and time to talk afterwards and so on.”

(Mother 6, boy 10mo)

Due to the restrictions during the COVID-19 pandemic, ongoing parent groups in primary CHC in Sweden were temporarily cancelled from March 2020. This affected the interviewed parents differently depending on the age of their infants at that time, where those who had planned to attend parent groups no longer were able to do so. While the affected parents understood and accepted the cancellations, many of them also expressed disappointment over not being offered any alternative support or guidance, such as online options to make contact with other parents, or tips on where to find relevant missed-out information.

“So, like this, now that we’re talking about it now, you feel like this; really why did we not continue at child healthcare with these parent groups, but digitally?”.

(Mother 9, boy 7mo)

### 3.7. Theme 7: INFANT for a Swedish Context

When INFANT was described, many parents recognized several advantages, such as: the workshops being led by trained health professionals, and information that is trustworthy and evidence-based. They also believed that offering the program through primary CHC would increase reliability and make parents understand its value.

“For me, it [running the program through primary CHC] would give me more security. It is not that I do not trust it otherwise, but rather that like this: that it [the program] is part of what I need as a parent.”

(Mother 4, boy 12mo)

Furthermore, when group sessions (such as in INFANT) were discussed, the interviewees further highlighted advantages, such as: being able to exchange experiences, hear others’ reasoning and discuss issues. It was also pointed out that some parents might ask questions that others do not dare to ask themselves or only realize its significance once the question has been brought up.

“Yes, questions that you yourself have thought of but may not come up with right then, they ask [other parents]. Or questions that you may have and do not dare to ask, so … It is always like that.”

(Mother 13, girl 7mo)

Finally, all parents were very positive about INFANT when it was described to them. Many of them had expressed a wish for more continuous support and to be able to find answers whenever questions arose. Therefore, they recognized INFANT as a good way of obtaining more information without having to ask someone for it and liked the idea of having it easily available in an app. Furthermore, they considered the combination of workshops (group sessions) with an app to be “perfect” as they complement each other. More specifically, the workshops would provide opportunities for peer support and contact with health professionals, whereas the app would provide accessible information at any time. Overall, the parents believed a program similar to INFANT would be very beneficial for making valuable information more easily available to new parents, and a much-needed complement to the support provided in primary CHC today.

“… It is tips and advice. That’s what you want. It’s that simple. // I know what is healthy and unhealthy. It’s more ‘how do I implement it and how much of it’ and all that.”

(Father 1, boy 10.5mo)

“This way … you don’t need to be afraid to lose a paper or forget something that they have said at the child healthcare center as well, because you have the app as support.”

(Mother 4, boy 12mo)

## 4. Discussion

This study sought to investigate Swedish parents’ experiences regarding feeding of solid foods, screen time, and physical activity; investigate parents’ needs and their perceptions of what information and support is provided in primary CHC; and explore whether a parenting program focused on child diet and active play would be relevant and utilized by Swedish parents. A total of seven themes were identified: (i) Feeling excited to enter a new phase; (ii) Parents’ responsibility of doing it “right” can be stressful; (iii) Motivated to learn during specific time windows; (iv) Information to trust; (v) The importance of social support from peers; (vi) Experiences of support received from CHC; and (vii) INFANT for a Swedish context. Overall, parents were excited to begin introducing food to their infants; however, this came with stress and uncertainty about whether they were doing it “right”. Interestingly, many parents stated that they are very motivated to learn, but this learning needed to happen in the specific time frames for when they felt they needed it. Furthermore, parents sought more information and support regarding food and feeding than they currently receive, and the majority felt they would welcome additional support around these issues from their primary CHC. Parents also highlighted the need for evidence-based information and the importance of social support from peers. Finally, Swedish parents were very positive about the prospects of engaging in a primary CHC delivered program to support them to promote healthy lifestyle behaviors from birth and felt this could compliment the care currently provided.

Overall, parents in this study felt stress regarding making the “right” choices for their infant, especially concerning the introduction of solid foods, and felt responsible for ensuring their child’s needs where met in terms of nutrients and amount of food. A study in Danish parents also found that parents strived to be perfect and responsible when transitioning from a milk-based diet to solid foods and these demands created insecurity and concern [24]. Similarly, a Swedish study described how parents of infants considered food as highly important to “child health” and perceived feeding issues as something particularly worrying [25]. In the present study this stress came with an expressed need for support from primary CHC and parents wished for additional support beyond what is currently provided. These findings provide early support for the consideration of a program such as INFANT which could provide additional time to focus on early life feeding and active play with CHC experts, and extend the CHC visit beyond the home setting with the app.

One of the main findings of this study was that parents are very motivated to learn; however, this appears to be limited to specific time windows when issues are or soon to be of direct relevance to them. Previously, it has been found that parents (and parents to be) are most receptive during pregnancy and the first years of life [26]. However, to the best of our knowledge no study has investigated at what time points parents want to receive certain information. This type of information is important in understanding when and under what circumstances parents of infants are receptive to health promotion information and parenting support. Knowing these “teachable moments” for health behavior change is valuable for successful intervention planning [27]. With regards to food introduction, according to Swedish guidelines very small tasting portions can begin from four months of age, if the infant is showing an interest in food [19]. All 20 parents in the present study were excited to start with tasting portions at four months of age. However, they stressed the need for support regarding food introduction at four months of age or slightly before, within a quite narrow time window. Thus, this clearly demonstrates the need for regular and adaptive support for parents over their child’s first year to capture these windows of opportunity to support parents. INFANT was designed to start at four months of age as Australian guidelines state food introduction should begin around six months of age [28], thus for the Swedish context it may need to start earlier (e.g., around three months) to capture this teachable window. Furthermore, INFANT uses the anticipatory guidance approach, which focuses upon preparing and informing parents on what to expect and how to cope with different behaviors or issues that may arise before they are needed [15]. Thus, this fits very well with the expressed needs of parents in this study.

Parents in this study emphasized the importance of peer support in order to exchange experiences and information with other parents going through similar situations. A recent study of Belgian mothers also found that there was a strong need to share with other mothers in order to compare experiences [29]. In the present study those without pre-established networks (e.g., friends or family members with a young child) often sought new contacts through parental groups offered through primary CHC. While parent groups are a universal service offered in Sweden [19], not all parents engage, with only 36% of parents attending at least one parental group session in Stockholm County in 2018 [2]. For those who attended parental groups in the current study, some parents reported differences regarding the level of support and information provided. As peer support has been reported to be extremely important, a program such as INFANT may be able to bridge this gap by aiding CHC nurses in creating evidence-based structured group sessions covering topics that Swedish parents want. Furthermore, it could aid in creating more equality between parental groups and possibly reduce the pressure placed on CHC nurses through reducing the number of extra visits booked by parents to discuss topics such as food introduction. Anecdotal evidence from the scale up of INFANT in Victoria, Australia has indicated that there were fewer incidental visits booked with the CHC nurse after INFANT was introduced (unpublished data).

Data collection for the present study occurred during the COVID-19 pandemic, thus some of the parents in the current study were affected by the cancellation of parental groups. Even though the parents understood why the groups needed to be cancelled, they still wanted alternative forms of support. The importance of support for mothers was highlighted in a study by Slomian et al. [29] who found that mothers with young infants often felt isolated. COVID-19 has brought upon increased isolation and has possibly created inequality within CHC in Sweden through the cancellation of parental groups. As not all women have social networks through family or friends, it is important that alternative support systems are created in order to fill this void. In the present study, while parents expressed they would prefer in-person contact with primary CHC, they were positive about the opportunity to participate in online workshops when this was not possible. Programs with structured sessions such as INFANT have the ability to be delivered over a secure online platform providing parents with the information they want, but also to provide the social support from their peers when in-person sessions are not possible. It is important to note that INFANT was successfully delivered online during the COVID-19 lockdown in Victoria, Australia.

In the present study only four parents recalled their CHC nurse discussing screen time with them. This is concerning as a recent systematic review found that the relationship between screen time and motor and cognitive development, adiposity, and psychosocial health to be either unfavorable or of no benefit [30]. Yet, many parents are unaware of potential negative consequences of screen time for young children. In a previous study, far fewer parents of infants identified negative aspects of screen time, whereas positive aspects, often incorrectly ascribed, were commonly identified (e.g., educational benefits) [31]. According to Radesky et al. [32] pediatricians play an important role regarding communicating appropriate screen time use with parents. This aligns with findings from the current study where parents stated that screen time was a sensitive issue and thus this conversation should be initiated by a trusted source. In Sweden parents have the most contact with their CHC nurse and thus this information would be best communicated through them. However, the lack of discussion around screen time in primary CHC is likely due to the fact that no concrete guidelines were available in Sweden until the World Health Organization released integrated global guidelines on physical activity, sedentary behavior, and sleep for children under five years of age in April 2019 [33]. Furthermore, a study by Laws et al. [34] found that Australian CHC nurses expressed hesitation around raising discussions regarding screen time as they feared parents may not be receptive to the information and it would affect their rapport with the parents. Therefore, this is another possible reason why so few parents in the current study reported having conversations with their CHC nurse regarding screen time. With regards to physical activity, parents in this study considered their children as “naturally active”, a finding reported in another study [31]. Physical activity in this study was not a concern for parents at the present time of the interview. However, many parents mentioned they would like information and advice from primary CHC about how to stimulate their child’s development. As CHC nurses may feel they do not have enough expertise to discuss physical activity and screen time, evidence-based programs such as INFANT could fill this knowledge gap and provide a valuable resource to build their confidence and support their work.

In the present study, even though the interviews included questions regarding food, physical activity, and screen time the majority of parents tended to focus their discussion around food. This was probably due to the fact that food was a concern for parents now, where they perceived physical activity and screen time as possible issues in the future. Multi-component programs like INFANT starting in early infancy are advantageous as they help parents with situations that they are currently experiencing as well as prepare them for future situations in order to build healthy lifestyle behaviors. INFANT or other similar programs have the potential to reach a wide variety of families as lifestyle behaviors impact all families. However, it is important to note that certain adaptions may need to be made if children have a disease/disorder or are not typically developing.

A strength of this study is the qualitative design which is very valuable for eliciting rich information regarding belief’s and personal experiences as well as for adapting interventions for new contexts [35]. During the analysis inductive thematic saturation was reached where no new codes or themes were found [36]. The trustworthiness of this study was strengthened by the use of an interview guide and by the engagement of researchers with various backgrounds systematically analyzing the data [37]. A limitation of this study is the use of convenience sampling to recruit participants and the fact that four of the participants were known to the interviewer. Initially, recruitment for this study was planned to be conducted through primary CHCs; however, with the onset of COVID-19 and subsequent restrictions this was not possible. A strength of the recruitment approach was that a broad group of parents were represented with participants coming from two geographically distinct areas in Sweden, representing attendance at 18 different primary CHCs, and all parents had different CHC nurses. The majority of parents (70%) had a university/college education, two parents (10%) were born outside of Sweden, and four (20%) of the participants were fathers. While qualitative samples are typically small and not designed to be representative of a population, future studies including a broader cross section of the Swedish population (e.g., different cultural backgrounds and levels of socioeconomic position), as well as including parents of children who have disabilities may provide additional richness to the results of the study. However, the strong consistent themes that were evident in this study, highlight the need for more information and support for parents during certain developmental periods (e.g., introduction of solid foods), which is likely to apply to all Swedish parents given the common developmental experiences. Finally, future research investigating Swedish CHC nurses’ needs and opinions regarding the implementation of INFANT or a similar program into primary CHC needs to be conducted before an efficacy trial can be performed.

## 5. Conclusions

This study found that parents of infants are excited to enter new phases in their child’s development, such as introducing solid food. However, entering new phases came with worry if they were doing things “right” and this led parents seeking more information and support. Overall, Swedish parents were very positive towards INFANT and felt that a program like that could provide them with the additional support they want and complement the care that is currently provided by primary CHC. Finally, the implementation of an evidenced-based program into primary CHC in Sweden has the potential to positively impact young children’s health through aiding parents to establish healthy lifestyle behaviors and aid in the prevention of overweight and obesity.

## Figures and Tables

**Table 1:** Descriptive characteristics of the participating parents and infants (*n* = 20).

Infants	Mean ± SD, *n* (%)	Minimum–Maximum
Age (months)	8.3 ± 4.0	2.0–14.8
Sex (*n*, %)		
Male	12 (60.0%)	
Female	8 (40.0%)	
**Parents**		
Age (years)	32.2 ± 3.2	27.4–39.2
Weight (kg)	67.3 ± 9.9	52.0–95.0
Height (cm)	168.3 ± 8.2	155.0–187.0
BMI (kg/m^2^) ^1^	23.7 ± 2.8	20.3–30.3
Sex (*n*, %)		
Male	4 (20.0%)	
Female	16 (80.0%)	
Education (*n*, %)		
High school	6 (30.0%)	
University/college	14 (70.0%)	
Place of birth (*n*, %)		
Sweden	18 (90.0%)	
Other	2 (10.0%)	
First time parent	17 (85.0%)	
Two parent household	20 (100%)	

Abbreviations: SD, Standard deviation; BMI, Body mass index. ^1^ Calculated using self-reported weight and height.

## Supplementary Materials

The following are available online at https://www.mdpi.com/2072-6643/12/12/3823/s1, Scheme S1: COnsolidated criteria for REporting Qualitative research (COREQ) checklist; Table S1: Core questions asked to the participating parents.
