# Supplementary material for: The Need for an Evidence-Based Program in Sweden to Support Parents to Create Healthy Lifestyle Behaviors from the Start of Life—Parental Perceptions
Source: Nutrients. 2020 Dec 14;12(12):3823. doi: 10.3390/nu12123823 (PMC7765081; doi:10.3390/nu12123823)
Supplement: Supplementary file 1 [file nutrients-12-03823-s001.zip › nutrients-1025201-suppl/nutrients-1025201-Suppl Table.pdf]

Table S1. Core questions asked to the participating parents.

Topic 1: Parents' experiences with introducing food to their infant	
1.	How old is your son/daughter today?
2.	Describe how you feel (or felt) before you start (started) introducing food to your child?
Topic 2: Roles and responsibilities regarding children's food and movement behaviors	
3.	How do you view your role and responsibilities as a parent regarding your child's diet?
4.	How do you view your role and responsibilities as a parent regarding your child's physical activity, sedentary behavior, and screen time?
5.	How much do you think you can influence your child's future eating and movement behaviors (e.g., physical activity, sedentary behavior)?
Topic 3: Information and support that parents received or would have liked to receive	
6.	If you are wondering or thinking about something regarding your child and food, where do you look for answers to your questions?
7.	With regards to breastfeeding and bottle feeding what is your opinion/experience on the information and support you have received from child primary healthcare?
8.	What is your opinion/experience regarding the information and support you have received from primary child healthcare regarding the introduction of food?
9.	What is your opinion/experience regarding the information and support you have received from primary child healthcare regarding physical activity and active play?
10.	What is your opinion/experience regarding the information and support you have received from child primary healthcare regarding sedentary behavior and screen time?
11.	Is there any special information or support that you lacked from child primary healthcare (which we have not already discussed)?
Topic 4: Adaption of program such as INFANT for a Swedish context<br>*Before the questions for theme 4 the parent received more background information regarding INFANT. This short summary consisted of how the program was set-up (i.e., four group sessions provided through primary child healthcare complemented by a mobile app) and that it begins when the infant is around four months of age*	
12.	What do you think about this type of program (with workshops) being offered here in Sweden?
13.	What is your opinion about receiving this information/support through a mobile application?
14.	We have considered two different types of approaches: (i) either four workshops/group meetings when the child is 6, 9, 12, and 15 months and at the same time have access to a mobile application or (ii) only have the mobile application without any workshops/group meetings. Which would you prefer?
15.	Do you have any other suggestions or ideas for other ways in which we could provide information and support regarding infant's food and activity to parents?
16.	In the future if we were able to offer a new parental support program regarding breast/bottle feeding, food introduction, physical activity, and screen time at what time point do you think we should invite families to the program?
17.	What type of information (e.g., different topics, information etc.) do you feel as a parent to a young child is important to provide?
18.	Do you have any other thoughts, opinions, or ideas that you would like to talk about?
